# A versatile method to fingerprint and compare the oxidative behaviour of lipids beyond their oxidative stability

**DOI:** 10.1038/s41598-023-34599-6

**Published:** 2023-05-19

**Authors:** Silvia Pizzimenti, Luca Bernazzani, Celia Duce, Maria Rosaria Tinè, Ilaria Bonaduce

**Affiliations:** 1 Department of Chemistry and Industrial Chemistry, University of Pisa, Via Giuseppe Moruzzi 13, 56124 Pisa, Italy; 2 Istituto Nazionale di Ottica (INO) - SS Pisa, CNR area di Pisa, Via Moruzzi 1, 56124 Pisa, Italy

**Keywords:** Chemistry, Materials science

## Abstract

In this work we propose the use of isothermal thermogravimetry to evaluate the oxidative stability of a lipid and to evaluate how the glyceride composition affects the entire oxidative process, to quantify the oxidation undertaken by the lipid, and numerically compare the oxidative behaviour of different lipids. The innovative aspect of the present method lies in the acquisition of a prolonged “oxygen uptake” curve (4000–10,000 min) of a lipid under oxygen and in the development of a semi-empirical fitting equation for the experimental data. This provides the induction period (oxidative stability), and allows to evaluate the rate of oxidation, the rate and the magnitude of oxidative degradation, the overall mass loss and the mass of oxygen taken by the lipid upon time. The proposed approach is used to characterize the oxidation of different edible oils with different degrees of unsaturation (linseed oil, sunflower oil, and olive oil) as well as chemically simpler compounds used in the literature to model the autoxidation of vegetable oils and lipids in general: triglycerides (glyceryl trilinolenate, glyceryl trilinoleate and glyceryl trioleate) and methyl esters (methyl linoleate and methyl linolenate). The approach proves very robust and very sensitive to changes in the sample composition.

## Introduction

Lipids, when exposed to atmospheric oxygen, are subject to a complex series of reactions which may evolve toward two main competitive pathways: oxidative degradation—resulting in the formation of polar species, including volatile compounds (such as short chain carboxylic acids, aldehydes and ketones), and cross-linking—resulting in the formation of higher molecular weight species^1^.

Oxidative degradation is one of the most common degradative pathways of lipids-based drug formulations^2,3^ and it leads to the so-called rancidity of lipid-rich food, which is characterised by the development of unpleasant smells, as well as a decrease in nutritional value^4^. In oil-based paints, a curing process dominated by oxidative degradation can negatively affect the painting stability and favour the rising of degradation phenomena upon time^5,6^. Polymerization reactions, on the other hand, are fundamental in the case of drying and semi drying oil-based paints, as they are responsible for the gradual conversion of the liquid paint into a solid painting layer^7^. Oxidative stability is also one of the most important quality parameters set by European and American standards for biodiesels^8^. The oxidative behaviour of a lipid is strictly related to its chemical composition in terms of the degree of unsaturation and the methylene bridge index (MBI: the mean number of bisallylic methylene positions), stereospecific positional distribution of fatty acids in the TAG molecules, lipid class (alkyl ester, triglyceride, phosphoglyceride, etc.), the presence of antioxidants and other trace components (enzymes, metals)^9^. In addition to the nature of the lipid, environmental factors (i.e. storage conditions), processing, and product formulations also affect its oxidation rate^9^. Several methods are routinely used to assess if an oil can be considered oxidized or not, and to rank the degree of stability to oxidation among different oils. Induction time, peroxide value, presence of free fatty acids and conjugated dienes and trienes, are commonly tested by means of automated methods, such as Rancimat (Metrohm, model 743, Herisau, Switzerland) and Oxitest—Oxidation Test Reactor (VELP, Usmate, MB, Italy), spectroscopy, and gas chromatography^10–13^. Alternatively, mass changes upon oxidation are also recorded, either using a scale before and after the oxidation event has taken place^14–17^, or using isothermal thermogravimetry (TG) under air flow^18–24^.

**Figure 1:**
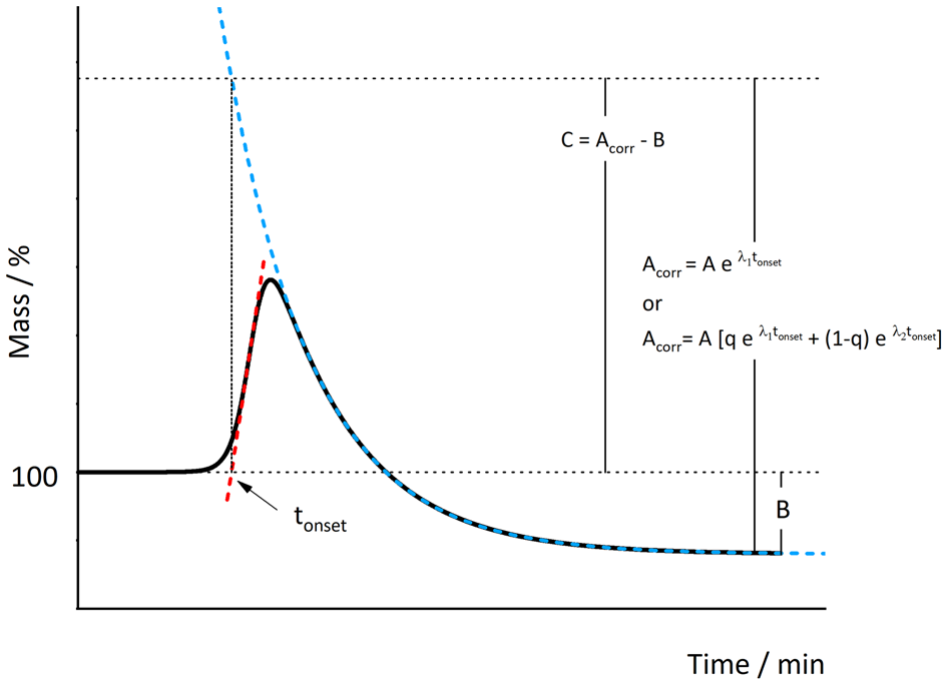
Exemplificative thermogravimetric curve. Schematic representation of the meaning of parameters *A*_corr_ for the *t*_onset_, *B* and *C* obtained with Eqs. (2a) and (2b).

During an Isothermal TG measurement, the sample is maintained at a constant temperature under a dry air/oxygen flow. The instrument output is a mass change as a function of time which is called “oxygen uptake” curve^18–25^. An exemplificative thermogravimetric curve representing an oxygen uptake profile of a plant oil is presented in Fig. 1. The presence of two regions is common to all the systems investigated^18–25^. The first is characterized by an increase in mass and the second by a mass decrease, which, with time, generally tends to a plateau. In the presence of antioxidants, an initial plateau is also observed, covering the time interval required for the mass uptake to be induced in the sample. In the region associated with the mass increase, peroxides formation is assumed to be the dominant reaction. In the region associated with the mass loss, oxidative degradation phenomena (leading to the formation of volatile compounds evaporating from the sample at the temperature of analysis) prevail. However, at any time, the mass change reflects a complex combination of the two aforementioned processes, as volatile products can be produced as soon as the first hydroperoxide decomposes^26^.

Generally, in the literature, the oxidative stability of a lipid, the presence of antioxidants, and the effect of pretreatments are associated exclusively to the induction period of oxygen uptake, and the latter is related to the onset time and onset temperature of mass gain. Consequently, oxygen uptake curves have frequently been acquired for relatively short times: only until the start of mass gain or until the start of mass decrease^18,19,23,24^*.* Also monitoring chemiluminescence^27,28^ or peroxide value^29,30^, which aims to evaluate lipid oxidation over time, takes into account only the first steps of oxidation.

In the present work we propose the use of isothermal thermogravimetry to evaluate the lipid oxidative stability and how the glyceride composition affects the entire oxidative process, to quantify the oxidation undertaken by the lipid, and to quantitatively compare the oxidative behaviour of different lipids.

The innovative aspect of the present method lies in the acquisition of a prolonged “oxygen uptake” curve (4000–10,000 min) and in the development of a semi-empirical fitting equation for the experimental data that allows: (i) to determine the induction time of the oxygen uptake process, (ii) to evaluate apparent time constants for the oxygen uptake process and for the main mass loss phenomena, (iii) to estimate the overall mass loss and the mass of oxygen taken by the lipid upon time, (iv) to project the trend of the curve for a time tending to infinite. The developed semi-empirical fitting equation for the oxygen uptake curve can be thus used to evaluate the stability towards oxidation and the kinetics of the process, estimate the tendency of the lipid towards oxidative degradation and crosslinking, and perform semiquantitative comparisons among different lipids.

In previous works^31,32^, we applied a first version of the semi-empirical model to fit the thermogravimetric profiles of four model paints prepared using different combinations of pigments and air drying oils.

In the present work, the fitting equation has been modified to make it better suited to efficiently describe systems with very different oxygen uptake profiles and thus different oxidative behaviours. To validate this new fitting method, the equation was used to study the oxygen uptake profiles of different lipid classes, edible plant oils, and lipid model systems, which differ for their PUFA (polyunsaturated fatty acids) content: olive oil, consisting of 60% of oleic acid, 12% of linoleic acid, and 1% of linolenic acid; sunflower oil consisting of 46% of linoleic acid, 30% of oleic acid, and 1% of linolenic acid; linseed oil consisting of 34% of linolenic acid, 30% of linoleic acid, and 11% of oleic acid^33^, glyceryl trioleate, glyceryl trilinoleate, and glyceryl trilinolenate; methyl linoleate; methyl linolenate; methyl oleate. The relative average amounts of fatty acids in the three oils were based on previously published data^33^. Pure triglycerides and methyl esters have been widely exploited in the literature to model the autoxidation of vegetable oils and lipids in general^34–52^.

## Results and discussion

### The fitting equation

To fit the experimental data of an oxygen uptake curve, a function was chosen which is a combination of a sigmoidal function $$\left(\frac{1}{1+{e}^{-{\lambda }_{0}\left(t-{t}_{0}\right)}}\right)$$ and an exponential or bi-exponential $$\left(A{e}^{-{\lambda }_{1}t}\right.$$ or $$\left.A\left[q{e}^{-{\lambda }_{1}t}+\left(1-q\right){e}^{-{\lambda }_{2}t}\right]\right)$$ decay function. The bi-exponential function was used whenever the fitting of the mass loss region carried out with a single exponential is not sufficiently accurate. The term *t*_0_ in the sigmoidal function, accounts for the delay observed in the mass increase and, therefore, correlates with the induction time in the peroxide formation process. The value of the time constant $${\lambda }_{0}$$ is correlated with the rate of the oxygen uptake. The time constant $${\lambda }_{1}$$, and when used $${\lambda }_{2}$$, are correlated with the rate of all phenomena leading to mass loss, $$q$$ and ($$1-q)$$ being the relative weight of the two exponentials, when both present. The choice of using exponential functions to describe both the oxygen uptake step and the loss of mass implicitly implies that the underlying kinetic processes are assumed to be of the first order or the pseudo first order with respect to the substrate concentration. This assumption is in accordance with what is reported in the literature for these experiments when carried out under non-limiting oxygen conditions^53,54^.

An analysis of experimental curves shown in previous works^31,32^ suggests that it should always be ${\lambda }_{0}\gg {\lambda }_{1},{\lambda }_{2}$, since the derivative $\left(\frac{dmass\%}{dt}\right)$, evaluated at the two inflection points, is always significantly greater, in absolute value, during mass gain versus mass loss. In these works^31,32^ we proposed the use of fitting functions of the type:

1a $$mass\%\left(t\right)=\frac{A{e}^{-{\lambda }_{1}t}}{1+{e}^{-{\lambda }_{0}\left(t-{t}_{0}\right)}}+B$$

or

1b $$mass\%\left(t\right)=\frac{A\left[q{e}^{-{\lambda }_{1}t}+\left(1-q\right){e}^{{-\lambda }_{2}t}\right]}{1+{e}^{-{\lambda }_{0}\left(t-{t}_{0}\right)}}+B$$

Since all exponentials cancel out when *t* approaches infinity, *B* represents the value of the function at the plateau. When *t* tends to zero, the function tends to $\left(\frac{A}{1+{e}^{{\lambda }_{0}{t}_{0}}}+B\right)$, therefore the mass% at time zero should be between $\frac{A}{2}+B$(when ${\lambda }_{0}{t}_{0}$ tends to zero) and B (when ${\lambda }_{0}{t}_{0}$ is large). Therefore, the two previously proposed functions can describe accurately oxygen uptake curves when the induction times are relatively short, with a small inaccuracy only in the initial part, easily disguised by the experimental error. In cases where ${t}_{0}$ (and consequently ${\lambda }_{0}{t}_{0}$) is large, the functions 1a and 1b tend instead to the constant value *B* both at the limit for $t\to \infty$ and for $t\to 0$, which makes the fitting clearly unacceptable in all those cases in which the experimental trend shows a step between the initial plateau and the asymptotic value for $t\to \infty$.

We therefore here propose the new following functions:

2a $$mass\%\left(t\right)=\frac{A{e}^{-{\lambda }_{1}t}-B}{1+{e}^{-{\lambda }_{0}\left(t-{t}_{0}\right)}}+100$$

or

2b $$mass\%\left(t\right)=\frac{A\left[q{e}^{-{\lambda }_{1}t}+\left(1-q\right){e}^{{-\lambda }_{2}t}\right]-B}{1+{e}^{-{\lambda }_{0}\left(t-{t}_{0}\right)}}+100$$

to accurately interpolate oxygen uptake curves independently from the value of t_0_. The functions (2a) and (2b) tend asymptotically to the value $100-B$ when $t\to \infty$, while for $t\to 0$ they acquire values in between $\frac{A-B}{2}+100$ and $100$. From the point of view of the interpretation of the fitting kinetic parameters, i.e. the time ${t}_{0}$ and the time constants ${\lambda }_{0}$, ${\lambda }_{1}$ and ${\lambda }_{2}$, nothing changes with respect to the fitting Eqs. (1a) and (1b). The same can be said about the meaning of the parameter *q,* which quantifies the relative weight of the two decreasing exponentials, when both are present. The meaning of *A* and *B* in Eqs. (2a) and (2b), however, is different. To summarize, the meaning of each parameter of the new equations, Eqs. (2a) and (2b), is as follows:

- ***A***: amplitude factor of the exponential function (or of the exponential functions) associated with the loss of mass.
- ***B***: deviation of the curve from 100% as *t* tends to infinity.
- **λ**_**0**_: apparent rate constant related to the mass increase.
- **λ**_**1**_ and **λ**_**2**_: apparent rate constants associated to the mass loss.
- ***q*** and **(1 − q)** (0 ≤ q ≤ 1): relative weights of the two relevant processes responsible for the mass loss.
- ***t***_**0**_: is the abscissa of the inflection point of the sigmoidal function, related to the induction time of the oxygen uptake.

To estimate the amount of mass of oxygen added, and the total mass loss due to oxidative degradation, we here introduce two more parameters, ${A}_{corr}=A{e}^{-{\lambda }_{1}t}$ (or ${A}_{corr}=A\left[q{e}^{-{\lambda }_{1}t}+\left(1-q\right){e}^{-{\lambda }_{2}t}\right]$) calculated at the *t*_onset_ and ${\varvec{C}}={A}_{corr}-B$. *t*_onset_ is the abscissa of the intersection point between the baseline and the tangent to the curve at the first inflection point. The meaning of parameters ***B*** and ***C*** and *A*_corr_ is exemplified in Fig. 1.

### Oxygen uptake profiles and data fitting

An oxygen uptake profile was recorded for each sample by isothermal thermogravimetric analysis under air flow. The shape of the curve has been proven to be affected not only by the oil composition, but also by external parameters, including the temperature of analysis^18,19,23,24^. Several efforts have been devoted in the literature to find the working temperature that allows to carry out the isothermal experiments in a relatively short time, without significantly affecting the reaction pathways with respect to those occurring at ambient temperature^18,19,23,24^. A wide range of temperatures, from 80 to 150 °C, has been investigated. In the analysis of vegetable oils, 80–90 °C are considered the best compromise between a faster oil oxidation rate and a reliable oxygen uptake curve^20,22,31^. In our work, thermogravimetric curves of plant oils and triacylglycerols were recorded at 80 °C. During preliminary experiments on methyl linoleate, the first step of mass increase was not visible at 80 °C and only a sharp decrease of mass was observed. For this reason, methyl linoleate and methyl linolenate were analysed at 60, 55, 50 and 40 °C. Methyl oleate was analysed only at 40 °C since already under this relatively low temperature only a slow mass decrease was observed within 8000 min of analysis (Fig. 3b).

### Oils, triglycerides, and methyl esters

**Figure 2:**
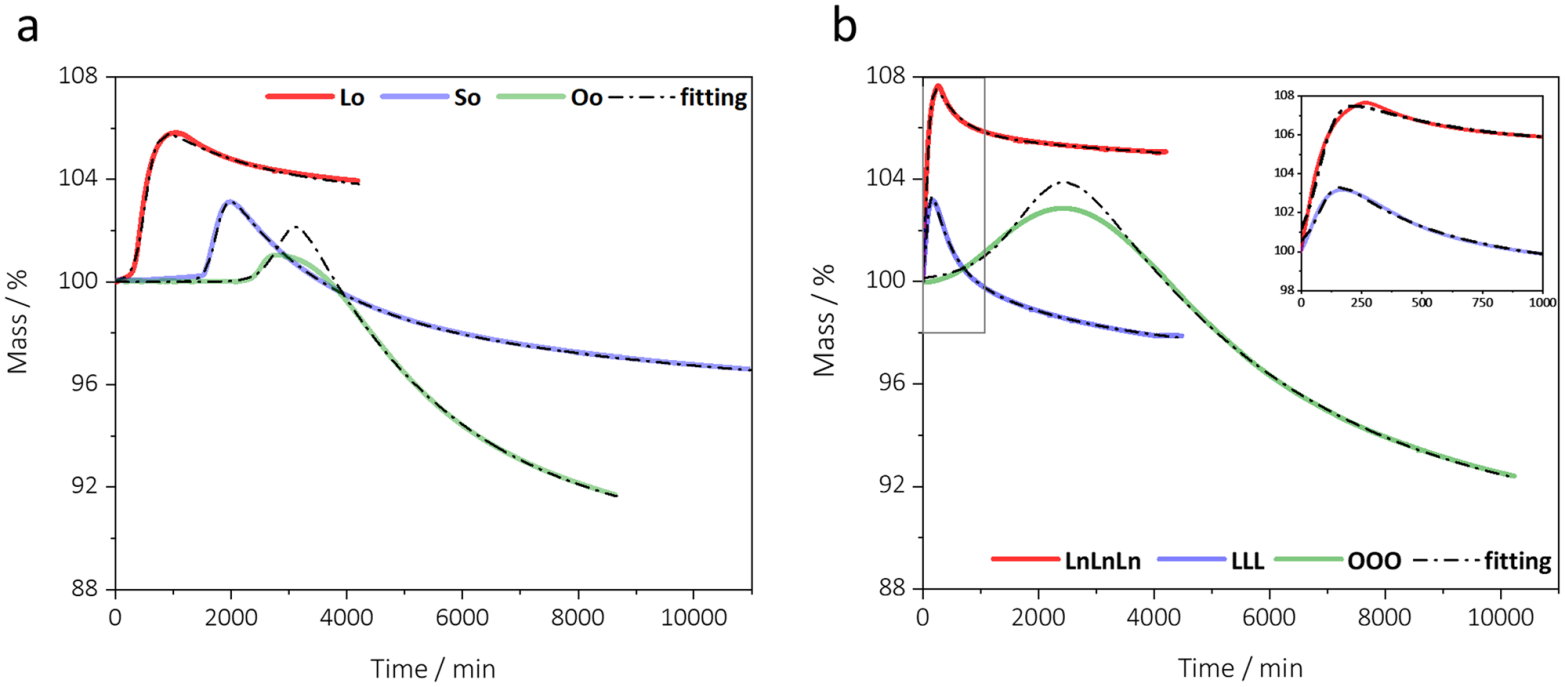
Thermogravimetric curves representing oxygen uptake profiles of (**a**) plant oils and (**b**) triacylglycerols obtained at 80 °C. Experimental (coloured solid line), and theoretical (black dash dot line) curves as obtained by Eqs. (2a) and (2b).

Figure 2a shows the experimental thermogravimetric curves and the fitting curves calculated by Eq. (2a) for plant oils (linseed oil, Lo, sunflower oil, So, and olive oil, Oo). Table 1 displays the curve-fitting parameters and their standard error, along with the relative χ^2^ and R^2^ coefficients, which indicate the quality of the fittings.

χ^2^ and R^2^ values for both linseed oil and sunflower oil are very good, but not as good for olive oil; for the latter the fitting equation is intrinsically unable to describe the experimental trend in the short interval between the end of mass increase and the beginning of mass loss, although it still perfectly describes the mass increase and the mass loss regions, and all the kinetics parameters show very small standard errors. Since in Oo the mass loss is delayed respect to the mass uptake, in this particular case, the parameter *C* (effective total oxygen taken by the sample) is the value of the maximum of the curve, while the total mass loss of the sample is the difference between the maximum and the end of the curve (A_corr_ calculated at *t*_endset_ that is the abscissa of the intersection point between the baseline before the mass uptake and the tangent at the inflection point of the mass decay).

**Table 1:** Values of parameters, χ^2^ and *R*^2^ coefficients obtained by fitting the experimental oxygen uptakes with Eqs. (2a) and (2b) for linseed oil (Lo), sunflower oil (So) and olive oil (Oo) and glyceryl trioleate (OOO) and with Eqs. (2a) and (2b) for glyceryl trilinolenate (LnLnLn) and glyceryl trilinoleate (LLL) at different temperatures.

System	*t*_0_	*t*_onset_	*A*	*A*_corr_	*B*	*C*	*q*	λ_0_ × 10^2^	λ_1_ × 10^3^	λ_2_ × 10^4^	χ^2^ × 10^3^	*R*^2^
	(min)	(min)	(%)	(%)	(%)	(%)		(min^−1^)	(min^−1^)	(min^−1^)		
Lo	521.9	512.8	3.9	3.3	− 3.5	6.8	–	1.0	0.5	–	9.3	0.995
So	1763.4	1468.9	21.4	8.8	6.1	2.7	0.8	1.0	0.6	0.6	1.1	0.999
Oo	2941.0	2464.0^a^	40.6	11.1^b^	0.1	1.1	–	0.6	0.4	–	2.4	0.999
LnLnLn	75.2	97.8	5.7	5.6	− 3.3	8.9	0.6	2.5	2.3	0.9	7.8	0.996
LLL	87.6	92.8	8.8	8.4	2.9	5.5	0.6	2.8	4.0	3.8	2.0	0.999
OOO	2340.0	649.1^a^	28.6	12.7^b^	9.7	3.1	–	0.2	0.3	–	14.1	0.999

The standard error of the parameters calculated are all less than 0.1.

^a^This is the value for *t*_*endset*_ and not *t*_*onset,*_.

^b^*Acorr* is calculated at *t*_*endset*_.

Figure 2b shows the experimental thermogravimetric curves and the fitting curves calculated according to Eq. (2b) for triacylglycerols, while Table 1 displays the curve-fitting parameters and their standard error, in brackets, along with the χ^2^ and R^2^ coefficients obtained. The similarity between the oxygen uptake curves of plant oils and those obtained for pure triacylglycerols, is rather evident. OOO shows the same short delay between the mass increase and the mass loss as Oo and the fitting equation describes well the mass increase and the mass loss regions but is inadequate to fit the small interval in between. *C* and *A*_corr_ for OOO are thus calculated as for Oo. *A*_corr_ and *C* parameter of the triacylglycerols are calculated in the same way as described above for the oils Table 1.

**Figure 3:**
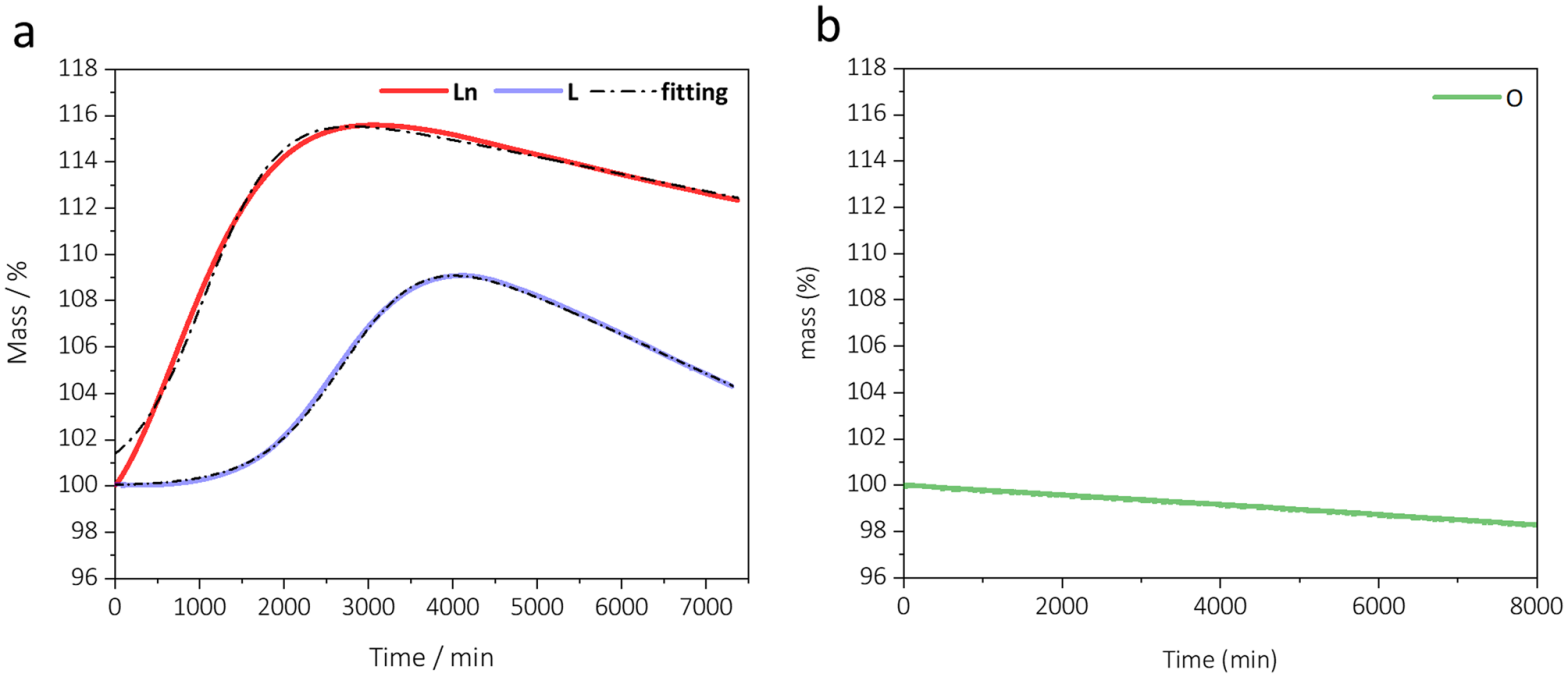
Thermogravimetric curves representing oxygen uptake profiles of (**a**) methyl linoleate (Ln) and methyl linoleate (L) at 60 °C, (**b**) methyl oleate (O) at 40 °C. Experimental (coloured solid line) and calculated (black dash dot line) curves as obtained by Eq. (2a).

Figure 3 shows the experimental and calculated curves for methyl linolenate and methyl linoleate, while the fitting parameters obtained at different temperatures are reported in Table 2. Although the general shape of the curves reflects quite well that of the corresponding triglycerides, it should be noted that the oxygen uptakes were measured at temperatures different than those of oils and acyl glycerides and therefore the fitting parameters must be compared with caution.

**Table 2:** Values of parameters and their standard error in brackets, χ^2^ and *R*^2^ coefficients obtained by fitting the experimental oxygen uptakes with Eq. (2a) for methyl linoleate and methyl linolenate at 40, 50, 55 and 60 °C.

System	Temperature of analysis	t_0_	t_onset_	A	A_corr_	B	C	λ_0_ × 10^2^	λ_1_ × 10^4^	χ^2^ × 10^2^	R^2^
		(min)	(min)	(%)	(%)	(%)	(%)	(min^−1^)	(min^−1^)		
Methyl linoleate	40 °C	3790	2444	n.d	n.d	n.d	n.d	0.2	1.1	1.6	0.998
		(1)						(< 0.1)	(< 0.1)		
	50 °C	1242	594	30	26	13	14	0.4	2.1	10.4	0.999
		(1)		(< 1)		(< 1)		(< 0.1)	(< 0.1)		
	55 °C	905	288	27	25	13	13	0.4	2.4	1.2	0.999
		(2)		(< 1)		(< 1)		(< 0.1)	(< 0.1)		
	60 °C	493	205	30	28	17	11	0.8	3.4	46.5	0.999
		(1)		(< 1)		(< 1)		(< 0.1)	(< 0.1)		
Methyl linolenate	40 °C	1114	341	n.d	n.d	n.d	n.d	0.2	0.6	6.8	0.998
		(1)						(< 0.1)	(< 0.1)		
	50 °C	702	192	18	18	3	15	0.3	0.8	4.2	0.994
		(1)		(< 1)		(< 1)		(< 0.1)	(< 0.1)		
	55 °C	476	112	16	16	1	14	0.5	1.9	2.7	0.997
		(1)		(< 1)		(< 1)		(< 0.1)	(< 0.1)		
	60 °C	369	100	18	17	3	14	0.6	2.6	257.3	0.999
		(< 1)		(< 1)		(< 1)		(< 0.1)	(< 0.1)		

By comparing the fitting parameters of the investigated systems with different PUFA content we can easily compare their oxidative behaviour as discussed in detail in the following paragraphs.

#### Induction time

*t*_0_ values reflect the trend of the oxidative stability of the lipid, and it has an inverse dependence on the number of unsaturations^41^. However, in plant oils, antioxidants play a major role in inhibiting or retarding the autoxidation process^55^. It is thus not straightforward to distinguish, in the oils, the contribution, or the extent of the effect of the antioxidants and the oxidative stability of the constituting glycerides. The oxidative stability because of the number and type of unsaturations is clearly visible when we compare the *t*_0_ values of acylglycerols which do not contain antioxidant.

#### Mass uptake

Comparing the fitting parameters *C* and λ_0_ related to the mass uptake, the observed trend is that linseed oil/glyceryl trilinolenate/methyl linolenate take more oxygen than sunflower oil/glyceryl trilinoleate/methyl linoleate which, in turn, take more oxygen than olive oil/trioleine (see Tables 1 and 2). The rate of autoxidation varies in the same order except for triglycerides for which λ_0_ is slightly higher for glyceryl trilinoleate than glyceryl trilinolenate, although λ_0_ of glyceryl trioleate is still the lowest. The trends of the parameter *C* is perfectly consistent with the number of double bonds present in the different materials, as the concentration of the primary oxidation products (hydroperoxides) as well as the maximum autoxidation rate increase with the concentration of unsaturations^56,57^. It also relates with the MBI index, that is the number of bis-allylic positions, as these are more susceptible to oxidation with respect to the allylic positions^37,41,58^. Linolenic, linoleic, and oleic acids present, respectively, two bis-allylic positions at C-11 and C-14, one bis-allylic position at C-11, and none. When we consider the numerical values, small differences are observed between the oils and the corresponding acylglycerols, which can be easily explained by considering the differences in their chemical composition, supporting the robustness of the fitting model. On the other hand, methyl esters are much faster to react and take up more oxygen. This different behaviour can be ascribed to the different mobility of the unsaturated tails in the two systems and to the higher viscosity of triglycerides compared to that of methyl esters which slow down the oxygen diffusion. These factors can both affect speed and pathways of reactions.

#### Mass loss

Oils with the greatest PUFA content produce the lowest amounts of secondary oxidation products, which comprise volatile species^59^. This is not only related to the number of unsaturations in general, but, again, to the number of bis-allylic positions available upon oxidation. Peroxyl radicals can abstract hydrogens only in bis-allylic positions, while alkoxyl radical may also abstract hydrogens in allylic positions^60^. As a result, the concentration of bis-allylic positions affects the reaction pathways: a high concentration of bis-allylic hydrogens favour propagation through radical abstraction, while addition of peroxides to double bonds is favoured in lipids with few bis-allylic hydrogens available^60^. The final result is that a high number of bis-allylic position favours cross-linking, while lipids with a low or null content of bis-allylic positions are more prone to oxidative degradation^6^. The fitting parameter *A* (amplitude of the decreasing exponential) is in line with this observation, showing that linseed oil/glyceryl trilinolenate/methyl linolenate is less prone to oxidative degradation with respect sunflower oil/glyceryl trilinoleate/methyl linoleate and olive oil/triolein, and that lipids based on oleic acid show the highest oxidative degradation.

As in polyunsaturated systems, in monounsaturated ones, peroxides and other oxidation intermediates are formed and they break down to a wide range of secondary oxidation products which include volatiles compounds^61^. Differently from oleic acid based oils and triglycerides, the experimental mass change of methyl oleate shows only a slow mass loss (Fig. 3b). Since this compound is not subject to crosslinking to a significant extent, it is possible to speculate that the evolution of volatile compounds occurs on the same timescale of the primary oxidation giving rise to a globally decreasing trend of the mass percent.

### Methyl linoleate and methyl linolenate at different temperatures

The shape of oxygen uptake curves does not only and unilaterally relate to the composition of the fatty acid substrates but also to reaction conditions and in particular to the temperature of analysis, which necessarily influences the reaction pathways^18,19,23,24^. As methyl esters are more reactive than triglycerides^9^, they were selected to investigate the effect of temperature on the oxidative behaviour of polyunsaturated lipids, by carrying out isothermal thermogravimetry at different temperatures (40, 50, 55 and 60 °C).

**Figure 4:**
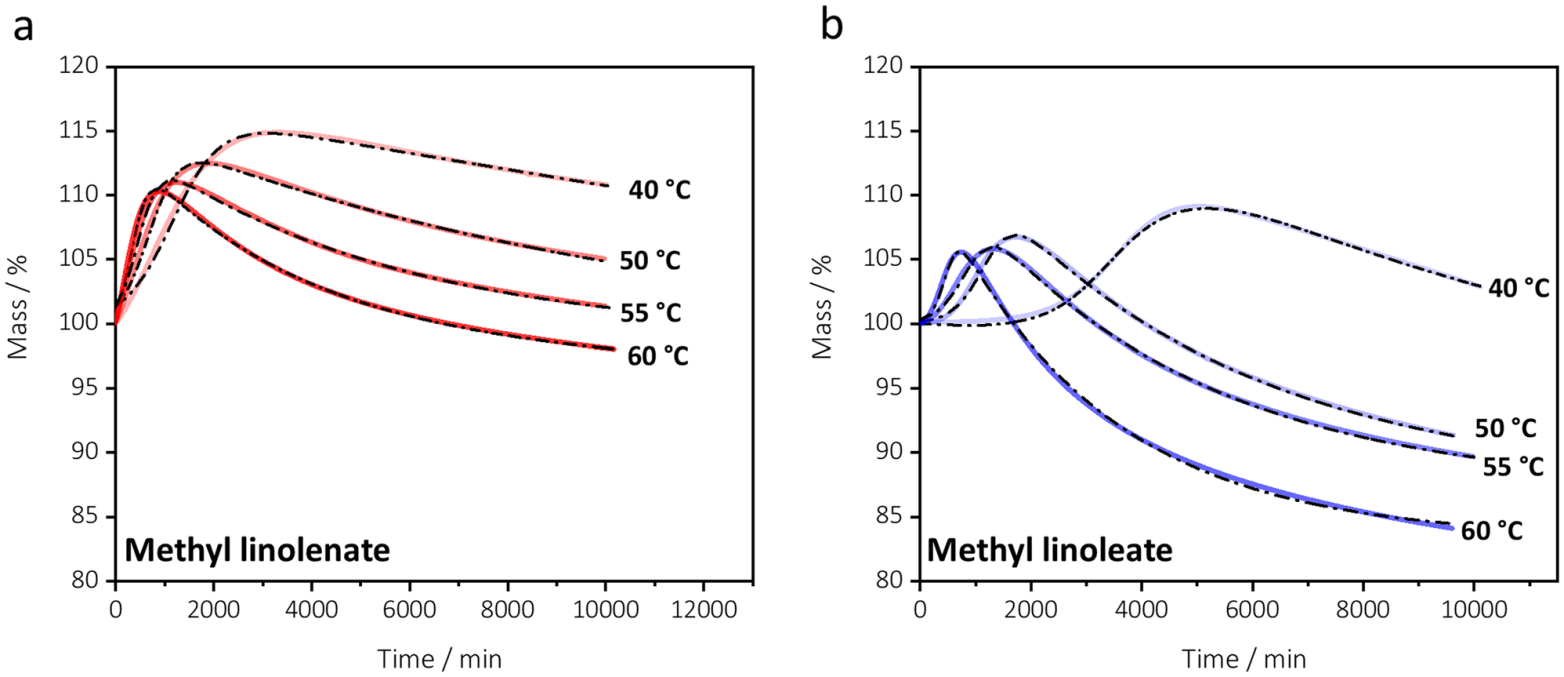
Thermogravimetric curves representing oxygen uptake profiles of (**a**) methyl linolenate and (**b**) methyl linoleate at 40, 50, 55 and 60 °C. Experimental curves are coloured solid line; theoretical curves obtained by Eq. (2a) are represented by black dash-dot lines.

Figure 4 shows the experimental and fitting curves [obtained from the fitting Eq. (2a)] for methyl linoleate and methyl linolenate at 40, 50, 55 °C along with those at 60 °C, already discussed, for comparison. The fitting parameters are displayed in Table 2.

The higher the temperature of analysis, the lower the maximum reached by the mass %. This can be related to the rate of decomposition of hydroperoxides. Richaud et al.^28^ carried out a study on the oxidation of methyl esters of oleic, linoleic, and linolenic acids based on chemiluminescence intensity data over time. The authors observed that the maximum of the curves of chemiluminescence intensity increases with temperature. The chemiluminescence signal is mainly related to the rate of hydroperoxides bimolecular decomposition: the higher the temperature, the higher the decomposition rate. The increase of hydroperoxides decomposition observed at higher temperatures promotes oxidative degradation, resulting in a lower mass % at the maximum of the curves.

Curves obtained at the different temperatures (40, 50, 55, and 60 °C) can be fitted with Eq. (2a).

The fitting of experimental data at 40 °C is quite unsatisfactory, though. This is because the experimental measurement was stopped when the descent had not yet assumed a decreasing exponential trend. For this reason parameters *A*, *B* and *C* are not reported in Table 2. Nevertheless, considerations on the mass increase among the four temperatures (40, 50, 55 and 60 °C) are still possible. The higher the temperature of analysis, the smaller are the values of *t*_0_ and *t*_onset_ and the higher are the values of the rate constant λ_0_. This is in agreement with previous work that showed that, when increasing the temperature of analysis, the induction time becomes smaller^25^, while the rate of hydroperoxides formation increases^29^. When the temperature rises (see parameters obtained at 50, 55 and 60 °C—Table 2), the parameters that estimate the total loss of mass and the amount of oxygen taken up, i.e. *A*_corr_ and *C*, differ little. Indeed, a slight increase of the effective loss (*A*_corr_) is observed, while the amount of oxygen taken up (*C*) drops down.

An Arrhenius plot can be built using the values of *λ*_0_ at different temperatures, to estimate the apparent activation energy of the oxygen uptake process. The logarithm of *λ*_0_ decreases linearly with the reciprocal of temperature for both methyl esters (R^2^ = 0.9197 and 0.9759 for methyl linoleate and methyl linolenate, respectively). The activation energy (*E*_a_ = – slope⋅*R*) of the uptake of oxygen was evaluated: *E*_act_ (methyl linoleate) = 55.6 ± 11.6 and *E*_act_ (methyl linolenate) = 44.2 ± 4.9 kJ mol^−1^. Methyl linolenate has an activation energy for the uptake of oxygen lower than methyl linoleate. The values are consistent with literature data obtained by chemiluminescence intensity data^28^ and gas-chromatography^62^ and data from non-isothermal DSC curves for ethyl linolenate and ethyl linoleate^37,63^.

Comparing the *A*, *A*_corr_ and *C* parameters obtained at 50, 55 and 60 °C, it is quite clear that the higher the temperature of analysis, the lower the amount of mass increase because of oxygen addition, and the higher the mass loss. The higher mass loss observed at higher temperatures could have several possible explanations. At higher analysis temperatures the rate of evaporation of the compounds produced by oxidation will be greater. Moreover, at higher temperatures, one can expect a higher mobility of molecules in the sample, which could affect reaction pathways. Finally increasing temperatures of analysis might overcome activation barriers which are in place at low temperature.

### Glyceryl trilinoleate and glyceryl trilinolenate at 25 °C

**Figure 5:**
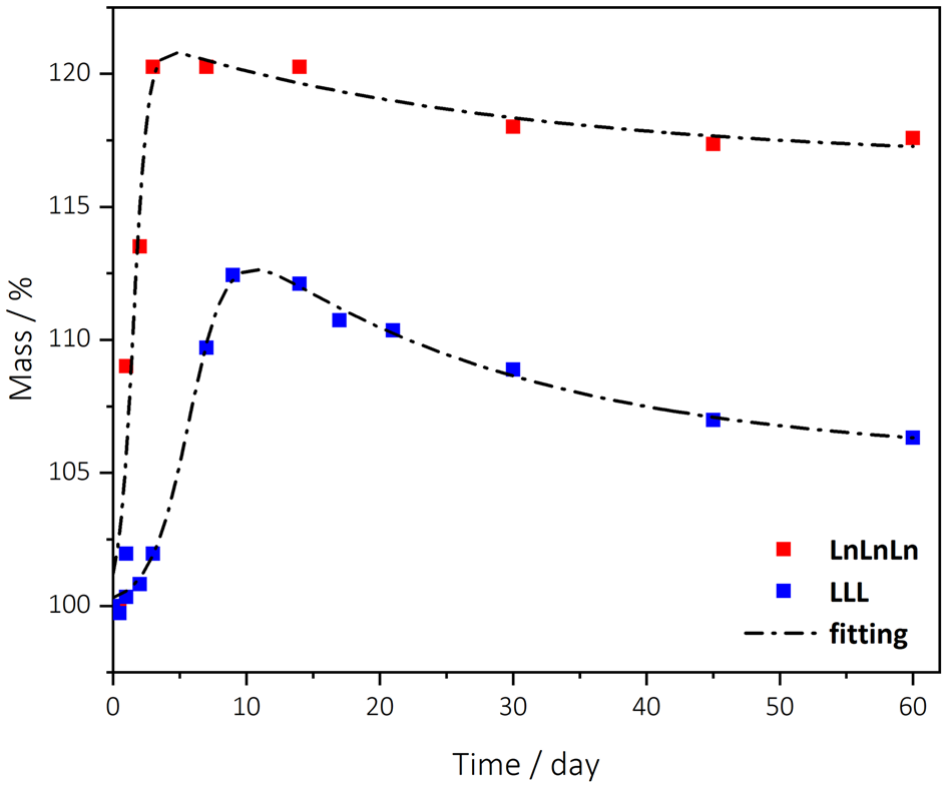
Oxygen uptake profiles of glyceryl trilinoleate (LLL) and glyceryl trilinolenate (LnLnLn) obtained at 25 °C. Experimental (coloured dot), and theoretical (black dash line) curves as obtained by Eq. (2a).

Based on the experiments carried out at different temperatures on methyl esters (paragraph 3.4), it is expected that kinetic parameters, and extent and rate of reaction with oxygen are different upon natural rather than accelerated analysis conditions. To visualize the extent of this difference, the experimental oxygen uptake profiles of glyceryl trilinoleate and glyceryl trilinolenate at 25 °C were recorded by weighing the same sample with a microbalance over a period of two months. The curves are shown in Fig. 5. The observed trends are qualitatively very similar to those recorded at 80 °C by TG. The experimental data obtained at 25 °C in natural ageing conditions were fitted with Eq. (2a) although in this case, given the small number of experimental points (about 10 points for each system), the statistical significance of the fitting parameters is less good than those obtained with the TG experiments. The curve-fitting parameters obtained are reported in Table 3. The most evident difference, obviously excluding the different time scale, is that, at room temperature, the mass increase % is higher than that at higher temperature, as already observed for methyl esters and reported in literatures for plant oils^23^. Another difference concerns the apparent rate constants related to the mass increase (*λ*_0_)_._ At 80 °C λ_0_ values are very similar for both triacylglycerols (2.5 × 10^–2^ and 2.8 × 10^–2^ for glyceryl trilinolenate and glyceryl trilinoleate, respectively), whereas at 25 °C, λ_0_ of glyceryl trilinolenate is twice than that of glyceryl trilinoleate (10^–3^ against 5 × 10^–4^).

**Table 3:** Values of parameters and their standard error in brackets, χ^2^ and R^2^ coefficients obtained by fitting the experimental oxygen uptakes with Eq. (2a) for glyceryl trilinolenate (LnLnLn) and glyceryl trilinoleate (LLL) obtained at 25 °C.

System	*t*_0_	*t*_onset_	*A*	*A*_corr_	*B*	*C*	λ_0_ × 10^2^	λ_1_ × 10^3^	χ^2^ × 10^2^	*R*^2^
	(min)	(min)	(%)	(%)	(%)	(%)	(min^−1^)	(min^−1^)		
LnLnLn	2217	597.4	5	4.8	− 17	21.5	0.1	0.025	4458.8	0.9611
	(< 1)		(4)		(5)		(< 0.1)	(< 0.001)		
LLL	8539	4357	12	10.8	− 6	16.4	0.05	0.032	330.4	0.986
	(< 1)		(2)		(1)		(< 0.01)	(< 0.01)		

### Comparison of the two model equations

As a further test, the new model equations were tested on polyunsaturated oils (linseed and safflower oil) already studied in our previous work^31^. TG curves of linseed and safflower oil, recorded under isothermal conditions at 80 °C were fitted according to the old Eq. (1b) and the new Eq. (2b). Table 4 reports the results of both fitting procedures. The numerical values obtained are generally perfectly consistent with each other, highlighting the same trends, thus leading to the same conclusions.

**Table 4:** Values of parameters and their standard error in brackets, Χ^2^ and R^2^ coefficients obtained by fitting the oxygen uptake profiles with Eqs. (1b) and (2b) for linseed oil (Lo), and safflower oil (So).

System	Eq	*t*_0_	*A*	*A*_*corr*_	*B*	*C*	*q*	λ_0_ × 10^2^	λ_1_ × 10^3^	λ_2_ × 10^4^	χ^2^ × 10^2^	R^2^
		(min)	%	%	%	%		(min^−1^)	(min^−1^)	(min^−1^)		
Lo	Equation (1b)	40.3	10.8	10.7	98.6	9.3	0.6	4.5	3.9	4.4	0.03	0.999
		(< 0.1)	(< 0.1)		(< 0.1)		(< 0.1)	(< 0.1)	(< 0.1)	(< 0.1)		
	Equation (2b)	47.1	10.6	10.5	1.6	8.9	0.6	5.6	3.7	4.0	0.007	0.999
		(0.1)	(< 0.1)		(< 0.1)		(< 0.1)	(< 0.1)	(< 0.1)	(< 0.1)		
So	Equation (1b)	26.4	12.6	12.0	95.3	7.3	0.7	2.9	6.3	6.2	0.15	0.999
		(0.1)	(< 0.1)		(< 0.1)		(< 0.1)	(< 0.1)	(< 0.1)	(< 0.1)		
	Equation (2b)	55.6	11.1	10.6	4.7	5.9	0.7	4.4	5.4	5.6	0.5	0.999
		(0.1)	(< 0.1)		(< 0.1)		(< 0.1)	(< 0.1)	(< 0.1)	(< 0.1)		

## Conclusions

The present work proposes semi-empirical fitting equations for the prolonged oxygen uptake curve of a lipid obtained by isothermal thermogravimetry. The aim is to obtain numerical parameters that describe the crucial steps of the oxidation process: the induction period, the rate of oxidation due to the formation of peroxides, an estimate of the amount of oxygen which reacts with the sample, and the rate and magnitude of oxidative degradation.

The fitting method is sensitive to the chemical differences between the samples, providing numerical parameters which can be related to the reactivity of different classes of lipids, characterized by different PUFA content and the MBI values, with oxygen.

The values that describe the rate and extent of reaction with oxygen depend strongly on the temperature of analysis. Data show that working in accelerated conditions at higher temperature, provides, in reasonable times of analysis, a quite good description of the oxidative behavior of a lipid. In general, though, the higher the temperature of analysis, the more oxidative degradation is observed. Above all, the method allows to obtain reliable comparisons among the oxidative behavior of different samples.

The equation model was thus used to describe and compare oxygen uptake curves of linseed oil (rich in linolenic acid), sunflower oil (rich in linoleic acid) and olive oil (rich in oleic acid), as well as triacylglycerols (glyceryl trilinolenate, glyceryl trilinoleate and glyceryl trioleate) and methyl esters (methyl linolenate, methyl linoleate, methyl oleate). The comparison between the curves obtained shows that triacylglycerols are very good models to describe the oxidative behavior of oils, excluding the influence of natural antioxidants present in an oil, which may vary considerably. Methyl esters, on the other hand, are more reactive than the corresponding triglycerides. So, although they are less suited than acylglycerols to mimic the oxidative behavior of plaint oils, especially from the kinetics point of view, they are significantly cheaper and better suited to molecular studies on the degradation mechanisms by mass spectrometry. The study performed on the oxidative degradation of methyl esters becomes particularly relevant due to their use as biofuels. We can conclude that the model to study lipid behavior must be chosen carefully, and that the proposed fitting equations represents a useful tool to guide this choice and support data interpretation.

## Materials and methods

### Samples

Linseed oil (Lo), sunflower oil (So) and olive oil (Oo), were purchased from a local market (Tuscany, Italy). Methyl linolenate (Ln), methyl linoleate (L), methyl oleate (O), glyceryl trioleate (OOO), glyceryl trilinoleate (LLL), and glyceryl trilinolenate (LnLnLn) were purchased from Sigma-Aldrich (purity ≥ 98%).

### Thermogravimetric measurements

Isothermal thermogravimetric analyses were carried out with a TA-Instruments thermo-balance, model Q5000IR for recording the oxygen uptake curve under a constant air flow (25 mL min^−1^). Linseed oil, sunflower oil, olive oil, glyceryl trioleate, glyceryl trilinoleate, glyceryl trilinolenate were analysed at 80 °C. Methyl linoleate and methyl linolenate were analysed at 40, 50, 55, and 60 °C, methyl oleate was analysed only at 40 °C. The sample masses ranged between 9 and 10 mg. Temperature calibration was based on the Curie point of paramagnetic metals. A multipoint calibration with five Curie points from reference materials (Alumel, Ni, Ni83%Co17%, Ni63%Co37%, Ni37%Co63%) was performed.

### Gravimetric measurements

Gravimetric measurements of samples held at ambient conditions (25 °C and 50% of relative humidity) were also registered over time by weighing oil samples (ca. 10 mg) put into an open aluminium pan with a Mettler Toledo balance AX 105; d = 0.01 mg/0.1 mg.

### Data fitting and elaboration

The fitting of thermogravimetric and gravimetric curves was performed with the software OriginPro8. Onset temperatures and derived parameters were calculated by Mathcad Prime 7.0.

## Data Availability

The data generated and analysed during this study which are not already included in this published article are available from the corresponding authors on reasonable request.

## References

[1] Schaich KM (2020). Bailey's Industrial Oil and Fat Products.

[2] Gabrič A, Hodnik Ž, Pajk S (2022). Oxidation of drugs during drug product development: Problems and solutions. Pharmaceutics.

[3] Musakhanian J, Rodier J-D, Dave M (2022). Oxidative stability in lipid formulations: A review of the mechanisms, drivers, and inhibitors of oxidation. AAPS PharmSciTech.

[4] Landers R, Rathmann D (1981). Vegetable oils: Effects of processing, storage and use on nutritional values. J. Am. Oil Chem. Soc..

[5] La Nasa J (2019). The role of the polymeric network in the water sensitivity of modern oil paints. Sci. Rep..

[6] Bonaduce I (2019). Conservation issues of modern oil paintings: A molecular model on paint curing. Acc. Chem. Res..

[7] Soucek M, Khattab T, Wu J (2012). Review of autoxidation and driers. Prog. Org. Coat..

[8] Botella L, Bimbela F, Martín L, Arauzo J, Sánchez JL (2014). Oxidation stability of biodiesel fuels and blends using the Rancimat and PetroOXY methods: Effect of 4-allyl-2, 6-dimethoxyphenol and catechol as biodiesel additives on oxidation stability. Front. Chem..

[9] Shahidi F, Zhong Y (2010). Lipid oxidation and improving the oxidative stability. Chem. Soc. Rev..

[10] Vecchio Ciprioti S, Paciulli M, Chiavaro E (2017). Application of different thermal analysis techniques to characterize oxidized olive oils. Eur. J. Lipid Sci. Technol..

[11] Frankel E (1993). In search of better methods to evaluate natural antioxidants and oxidative stability in food lipids. Trends Food Sci. Technol..

[12] Läubli MW, Bruttel PA (1986). Determination of the oxidative stability of fats and oils: Comparison between the active oxygen method (AOCS Cd 12–57) and the rancimat method. J. Am. Oil. Chem. Soc..

[13] Tinello F (2018). Comparison of OXITEST and RANCIMAT methods to evaluate the oxidative stability in frying oils. Eur. Food Res. Technol..

[14] Antolovich M, Prenzler PD, Patsalides E, McDonald S, Robards K (2002). Methods for testing antioxidant activity. Analyst.

[15] Shahidi F (2005). Bailey's Industrial Oil and Fat Products, Edible Oil and Fat Products: Processing Technologies.

[16] Niklová I, Schmidt Š, Habalová K, Sekretár S (2001). Effect of evening primrose extracts on oxidative stability of sunflower and rapeseed oils. Eur. J. Lipid Sci. Technol..

[17] Shahidi, F. & Zhong, H. J. Methods for measuring lipid oxidation. In *Bailey's Industrial Oil and Fat Products*, 1–27 (Wiley, 2005).

[18] Hassel R (1976). Thermal analysis: An alternative method of measuring oil stability. J. Am. Oil Chem. Soc..

[19] Buzás I, Simon J, Holló J (1977). Effect of the experimental conditions on the thermooxidative behaviour of vegetable oils. J. Therm. Anal..

[20] Lazzari M, Chiantore O (1999). Drying and oxidative degradation of linseed oil. Polym. Degrad. Stab..

[21] Bonaduce I (2012). A multi-analytical approach to studying binding media in oil paintings. J. Therm. Anal. Calorim..

[22] Tamburini D (2016). An investigation into the curing of urushi and tung oil films by thermoanalytical and mass spectrometric techniques. Polym. Degrad. Stab..

[23] Gao F, Birch J (2016). Oxidative stability, thermal decomposition, and oxidation onset prediction of carrot, flax, hemp, and canola seed oils in relation to oil composition and positional distribution of fatty acids. Eur. J. Lipid Sci. Technol..

[24] Mikula M, Khayat A (1985). Reaction conditions for measuring oxidative stability of oils by thermogravimetric analysis. J. Am. Oil. Chem. Soc..

[25] Torquato AS (2020). Kinetic parameters of the thermal oxidation and degradation reactions in soybean oil and palm olein. J. Braz. Chem. Soc..

[26] Frankel, E. N. Chemistry of autoxidation: Mechanism, products and flavor significance. In *Flavor chemistry of fats and oils*, 1–37 (Wiley, 1985).

[27] Neeman I, Joseph D, Biggley W, Seliger H (1985). Induced chemiluminescence of oxidized fatty acids and oils. Lipids.

[28] Richaud E (2012). Rate constants of oxidation of unsaturated fatty esters studied by chemiluminescence. Chem. Phys. Lipid..

[29] Du Plessis L, De Villiers J, Van Der Walt W (1985). Stability studies on methyl and ethyl fatty acid esters of sunflowerseed oil. J. Am. Oil Chem. Soc..

[30] Li X (2020). Kinetic models to understand the coexistence of formation and decomposition of hydroperoxide during lipid oxidation. Food Res. Int..

[31] Pizzimenti S (2021). Oxidation and cross-linking in the curing of air-drying artists' oil paints. ACS Appl. Polym. Mater..

[32] Pizzimenti S, Bernazzani L, Tinè MR, Duce C, Bonaduce I (2022). Unravelling the effect of carbon black in the autoxidation mechanism of polyunsaturated oils. J. Therm. Anal. Calorim..

[33] La Nasa J, Ghelardi E, Degano I, Modugno F, Colombini MP (2013). Core shell stationary phases for a novel separation of triglycerides in plant oils by high performance liquid chromatography with electrospray-quadrupole-time of flight mass spectrometer. J. Chromatogr. A.

[34] Cosgrove JP, Church DF, Pryor WA (1987). The kinetics of the autoxidation of polyunsaturated fatty acids. Lipids.

[35] Yamazaki M, Nagao A, Komamiya K (1980). High pressure differential thermal analysis (HPDTA) of fatty acid methyl esters and triglycerides. J. Am. Oil. Chem. Soc..

[36] Li J, Liu J, Sun X, Liu Y (2018). The mathematical prediction model for the oxidative stability of vegetable oils by the main fatty acids composition and thermogravimetric analysis. LWT.

[37] Litwinienko G, Kasprzycka-Guttman T (2000). Study on the autoxidation kinetics of fat components by differential scanning calorimetry. 2. Unsaturated fatty acids and their esters. Ind. Eng. Chem. Res..

[38] Issariyakul T, Dalai AK (2014). Biodiesel from vegetable oils. Renew. Sustain. Energy Rev..

[39] Dubrulle L, Lebeuf R, Fressancourt-Collinet M, Nardello-Rataj V (2017). Optimization of the vegetable oil composition in alkyd resins: A kinetic approach based on FAMEs autoxidation. Prog. Org. Coat..

[40] Serrano M (2014). Influence of blending vegetable oil methyl esters on biodiesel fuel properties: oxidative stability and cold flow properties. Energy.

[41] Knothe G, Dunn RO (2003). Dependence of oil stability index of fatty compounds on their structure and concentration and presence of metals. J. Am. Oil. Chem. Soc..

[42] Frankel EN (1991). Recent advances in lipid oxidation. J. Sci. Food Agric..

[43] Terao J, Matsushita S (1977). Geometrical isomers of monohydroperoxides formed by autoxidation of methyl linoleate. Agric. Biol. Chem..

[44] Berdeaux O (2012). A detailed identification study on high-temperature degradation products of oleic and linoleic acid methyl esters by GC–MS and GC–FTIR. Chem. Phys. Lipid..

[45] Coxon DT, Price KR, Chan HW-S (1981). Formation, isolation and structure determination of methyl linolenate diperoxides. Chem. Phys. Lipid..

[46] Frankel E, Neff W, Selke E, Weisleder D (1982). Photosensitized oxidation of methyl linoleate: Secondary and volatile thermal decomposition products. Lipids.

[47] Han IH, Csallany AS (2009). Formation of toxic α, β-unsaturated 4-hydroxy-aldehydes in thermally oxidized fatty acid methyl esters. J. Am. Oil. Chem. Soc..

[48] Neff W, Frankel E, Selke E, Weisleder D (1983). Photosensitized oxidation of methyl linoleate monohydroperoxides: Hydroperoxy cyclic peroxides, dihydroperoxides, keto esters and volatile thermal decomposition products. Lipids.

[49] Neff WE, Frankel EN, Fujimoto K (1988). Autoxidative dimerization of methyl linolenate and its monohydroperoxides, hydroperoxy epidioxides and dihydroperoxides. J. Am. Oil Chem. Soc..

[50] Terao J, Matsushita S (1975). Further oxygenated compounds produced from methyl linoleate monohydroperoxides at the process of autoxidation. Agric. Biol. Chem..

[51] Luna P, De La Fuente M, Salvador D, Márquez-Ruiz G (2007). Differences in oxidation kinetics between conjugated and non-conjugated methyl linoleate. Lipids.

[52] Vannoni L (2022). Disclosing the chemistry of oil curing by mass spectrometry using methyl linoleate as a model binder. Microchem. J..

[53] Hess P (1950). Oxidation of linseed oil. Ind. Eng. Chem.

[54] Labuza TP, Dugan L (1971). Kinetics of lipid oxidation in foods. Crit. Rev. Food Sci. Nutr..

[55] Kamal-Eldin A (2006). Effect of fatty acids and tocopherols on the oxidative stability of vegetable oils. Eur. J. Lipid Sci. Technol..

[56] Mallégol J, Gardette J-L, Lemaire J (2000). Long-term behavior of oil-based varnishes and paints: Fate of hydroperoxides in drying oils. J. Am. Oil Chem. Soc..

[57] Holman RT, Elmer OC (1947). The rates of oxidation of unsaturated fatty acids and esters. J. Am. Oil. Chem. Soc..

[58] Adhvaryu A, Erhan S, Liu Z, Perez J (2000). Oxidation kinetic studies of oils derived from unmodified and genetically modified vegetables using pressurized differential scanning calorimetry and nuclear magnetic resonance spectroscopy. Thermochim. Acta.

[59] Multari S, Marsol-Vall A, Heponiemi P, Suomela J-P, Yang B (2019). Changes in the volatile profile, fatty acid composition and other markers of lipid oxidation of six different vegetable oils during short-term deep-frying. Food Res. Int..

[60] Schaich KM, Shahidi F (2005). Bailey's industrial oil and fat products. Major Reference Works.

[61] Bonetti R, Parker WO (2019). Insights into polymerization of vegetable oil: Oligomerization of oleic acid. J. Am. Oil. Chem. Soc..

[62] Ishido E, Minemoto Y, Adachi S, Matsuno R (2001). Oxidation of linoleic acid and methyl linoleate mixed with saturated fatty acid or its methyl ester. LWT-Food Sci. Technol..

[63] Litwinienko G (2001). Autooxidation of unsaturated fatty acids and their esters. J. Therm. Anal. Calorim..

